# Experiences of Parent Coaches in an Intervention for Parents of Young Children Newly Diagnosed with Type 1 Diabetes

**DOI:** 10.3390/children11091036

**Published:** 2024-08-24

**Authors:** Carrie Tully, Manuela Sinisterra, Wendy Levy, Christine H. Wang, John Barber, Hailey Inverso, Marisa E. Hilliard, Maureen Monaghan, Randi Streisand

**Affiliations:** 1 Children’s National Hospital, Washington, DC 20010, USA; 2 School of Medicine and Health Sciences, The George Washington University, Washington, DC 20052, USA; 3 Department of Pediatrics, Baylor College of Medicine and Texas Children’s Hospital, Houston, TX 77030, USA

**Keywords:** diabetes, parenting, psychosocial, peer support

## Abstract

Objectives: This paper explores parent coaching experiences supporting parents of young children newly diagnosed with type 1 diabetes in a clinical trial. Methods: In a trial for 157 parents, those in the intervention arm (*n* = 116) were paired with a parent coach (n = 37; Mage = 37.9 years, SD = 3.9; 94.6% mothers, 81.1% White non-Hispanic). Parent coaches provided diabetes-specific social support. Parent coaches completed monthly surveys and satisfaction/feasibility surveys, with a subset (n = 7) undergoing qualitative interviews at the end of this study. Results: There were 2262 contacts between participants and their parent coaches, averaging 14.4 (SD = 9.3) per participant. Parent coaches reported that the most commonly used methods were text messages (67.9%) and emails (18.7%), with 33.6% having in-person visits. Coaches reported high satisfaction and belief in their usefulness to participants during the first 9 months after T1D diagnosis. Themes discussed by parent coaches about their experience in mentoring included relationship building, expertise sharing, personal growth, gratification, and intervention optimization suggestions. Conclusions: Parent coaching post T1D diagnosis involves regular, multi-method contacts. It is highly acceptable and valuable for parent coaches to mentor other parents of young children newly diagnosed with T1D.

## 1. Introduction

Type 1 diabetes (T1D) is a prevalent chronic condition in childhood, particularly among youth under age 20 [1]. T1D is commonly diagnosed in young and school-aged children [2]. Managing T1D in young children can be challenging due to behavioral and developmental factors [3], as well as medical contributors like increased insulin sensitivity or a honeymoon period [4].

Parents of children diagnosed with T1D undergo emotional adjustment and intensive education for T1D management [5]. Following diagnosis, many parents experience distress, fear, worry, self-doubt, and isolation [6,7,8]. Long-lasting parent stress and depression can negatively impact youth with T1D, leading to elevated glycemic markers and difficulties in self-management and emotional functioning [9,10,11,12].

Interventions for parents of young children following T1D diagnosis are essential to improve child and parent outcomes and mitigate health risks. A behavioral intervention that benefits children by focusing primarily on parents is justified by the social-ecological systems model [13], which posits that children’s health is influenced by multiple layers of their environment, with parents as the most proximal level of influence. By addressing parental behaviors and practices, interventions can create an environment that promotes better health outcomes for the children.

While existing behavioral interventions have focused on older children, there is a need to address the immediate post-diagnosis period within T1D [14,15,16]. Incorporating social support, such as peer coaching, can provide tools and education to effectively integrate T1D management into family life. Peer coaching interventions have been successful in adults with T1D [17,18].

Building on previous parent coaching interventions and pilot data [19,20,21], a behavioral intervention was developed and tested for parents of young children after T1D diagnosis, incorporating parent coaching. Parent coaches are more experienced parents of youth with T1D who are trained to offer peer support, use active listening skills, support self-efficacy, share their experiences, and suggest resources [20]. As part of a stepped-care behavioral intervention trial, First STEPS (Study of Type 1 in Early childhood and Parenting Support), parents of young children (age 1–6) newly diagnosed with T1D randomized to the intervention arm were paired with a peer parent coach for the 9-month intervention period in addition to other behavioral intervention components [22,23]. The primary aim of the parent study was to determine if families who participated in First STEPS had children with lower HbA1c values and parents with lower mood symptoms than families who received usual care. First STEPS improved parents’ moods [24]. However, the experience of being a parent coach remains unknown. This paper describes an exploratory, post hoc analysis of the activities and experiences of the parent coaches in the intervention.

## 2. Materials and Methods

The IRB-approved multi-site trial took place at two children’s hospitals in Washington, DC, and Houston, TX. The First STEPS study used a stepped-care intervention design involving three levels, or intensities, of treatment to families to achieve optimal outcomes [22,23]. The first “step” of intervention involved matching participants with the parent coach for the duration of the 9-month intervention period, which is the focus of the present paper. There were N = 157 families randomized; 116 were randomized to the stepped-care intervention and were matched with coaches. The parents were majority female (91.7%) with a mean age of 34.9 ± 7.0, and 87.3% were college graduates. Parents were 60.0% non-Hispanic White, 14.8% non-Hispanic Black, 14.8% Hispanic, 5.2% Asian/Asian American, and 5.2% multiracial. Their children had a mean age of 4.5 ± 1.6 and were 54.8% female. For full demographics, please see Hilliard et al., 2022 [22].

Parent coaches were recruited from the diabetes clinics at both medical centers on an ongoing basis. Providers nominated parents who effectively managed diabetes and could serve as positive role models for parents of newly diagnosed children. Eligible parents had a child diagnosed with T1D between the ages of 1 and 6, with no specific criteria regarding diabetes management or technology use. After sending letters to potential coaches, research team members followed up with phone calls to gauge interest and availability. Parent coaches were selected based on their warmth, understanding/compassion, and flexibility in diabetes management (see Tully et al., 2017 for a detailed recruitment description [23]).

We sent 86 recruitment letters to parents and screened 68 (79.1%) individuals. Out of those, 52 were considered eligible, and 45 remained interested. Ultimately, 37 coaches were trained and matched with participants. Ineligibility reasons included personal psychosocial stress, plans to relocate, and conflicts of interest due to parental jobs (e.g., physicians, employees of the funding agency). Some potential coaches experienced changes in availability, eligibility, or interest between the screening conversation and their scheduled training. For example, one coach did not complete the required online research ethics training, one coach’s family moved out of the study area, and one coach declined to be matched with a participant.

Parent coaches provided informed consent and completed baseline questionnaires on psychosocial health. In the intervention arm, participants received their parent coach’s contact information and were encouraged to initiate contact within the first week, primarily through email or phone. If possible, an in-person meeting was arranged. During the first 3 months of the intervention, coaches reached out once per week, followed by monthly contact for months 4–9, totaling 18 interactions. Parent coaches received a small gift card at the conclusion of their contact with each participant.

Parent coaches remained in the study until they requested to discontinue or were discontinued by the study team due to difficulties in contact. When coaches completed their involvement, they repeated psychosocial measures and filled out a satisfaction questionnaire. A subset of coaches (n = 7) participated in qualitative interviews to gather their experiences, aiming for diverse representation in demographics, trial length, and site.

Parent coaches underwent a 4 h training session and received monthly phone supervision with licensed mental health professionals from the study team. Coaches were compensated for their time in training and per participant. The training covered human subjects research, privacy protection, informed consent, interaction guidelines, role-playing mentorship skills, participant assignment, call structure, and supervision. The supportive role of parent coaches was emphasized, highlighting the distinction between their role and that of healthcare clinicians. Coaches were trained not to provide medical advice and to refer participants seeking diabetes management information to their medical team. Emergency contact information for medical or mental health crises was provided. Coaches’ interest in continued involvement was assessed before each new assignment. Matchings were based on coach availability, proximity, age of child at diagnosis, and personal characteristics when feasible (e.g., matching bilingual participants, gender/roles). Coaches were asked about their availability for additional participant matches before new assignments were given because some preferred to carry fewer participants during busy times in their own lives (e.g., back to school, job transitions). Because the protocol included encouraging coaches to meet in person with their participant at least once and each of the study location hospitals had large catchment areas, physical proximity was also attempted, though not always possible.

Parent coaches self-reported about their demographic characteristics and their child’s medical history. Monthly online surveys via REDCap (Research Electronic Data Capture) [25] collected data on parent coaches’ contact with assigned participants, including the number of overall contacts, phone calls, texts, emails, and intervention content areas covered. At the end of their participation, parent coaches completed a satisfaction survey (cronbach’s α = 0.78). Study participants in the intervention condition also reported their satisfaction with their contacts with the parent coach.

We conducted qualitative interviews with n = 7 parent coaches once their participation in the study was completed (mean age 38.14 ± 2.47 years; 3 from DC and 4 from Texas; 6 female mothers, 1 male father; 6 identifying as White, 1 identifying as Black; 5 as non-Hispanic/Latino and 2 as Hispanic/Latino; average age of child at T1D diagnosis = 3.57 ± 1.13; 5 on an insulin pump; 6 on CGM). There was purposeful sampling for interviews. We selected coaches from each study site with a range of ages of their own children at the time of T1D diagnosis, specific demographic criteria (e.g., race/ethnicity and gender), and different reasons for discontinuing the First STEPS project. Parent coaches worked with between 3 and 7 participants during their time in the study (mean = 4.29 ± 2.29), and two of the interviewed parent coaches discontinued the trial before enrollment was completed, due to their time limitations. The 30 min phone interviews were conducted by a trained member of the research staff with whom the parent coach had not worked directly. The interviews covered topics such as the program structure, experience as a parent coach, and any difficulties encountered. Interviews were audio recorded and transcribed.

The data analysis plan follows: We examined the descriptive characteristics of the demographic and psychosocial characteristics of the parent coaches. We reported the frequency, duration, and content of the parent coach contacts with parents, as well as participant and parent coach satisfaction with participation. We explored associations among parent coach demographics and duration in the project. For the qualitative interviews, the coders, their supervisors, and the study investigators met and came to consensus on the final codes and themes regarding data from interviews. Prior to analysis, transcriptions were reviewed by the research team to ensure accuracy. We used thematic analysis as the goal of analysis was to identify action-oriented themes emerging across the data set rather than unique features of individual cases [26]. We coded and analyzed the qualitative interview transcripts to identify themes related to parent coaches and experiences [27]. The following iterative steps were used. Four research staff across the two study sites were the primary coding teams (first author, psychologist; third author, social worker; sixth author, diabetes research coordinator; seventh author, diabetes psychologist). The authors first read a subset (n = 3) of transcripts multiple times, identified key concepts and definitions, and created lists of preliminary codes. The primary coding team each independently coded all transcripts and met weekly to resolve any discrepancies. If new concepts did not fit within existing codes, the teams met to discuss and edit the codebook (e.g., revise definitions, add new codes, collapse categories). The first author then sorted the codes into groupings of ideas to develop themes and subthemes to facilitate interpretation, which were reviewed with the study team to ensure relevance to the intervention.

## 3. Results

Of the 37 parent coaches, most were mothers (94.6%), married (83.8%), and college-educated (75.6%; see Table 1).

There was a variety of work schedules; a little less than half were working full-time (46%). The 37 parent coaches were assigned all the participants randomized to the intervention condition (n = 116) through the span of the trial. There was a median of three participants per parent coach, with a range of 2–7. Re-assignment of a participant to a new parent coach occurred in <2% of the cases, due to parent coaches discontinuing the trial. Coach marriage status was marginally associated with longer duration in the project (ρ = 0.33; *p* = 0.05); otherwise, coach full-time employment status, number of children, child age, and income were unrelated to coach duration in the project (*p* > 0.05).

There were a total of 2262 contacts between parent coaches and participants in the form of phone calls, text messages, emails, and in-person meetings throughout the 9-month intervention duration. There was a mean of 14.4 (SD = 9.3) contacts between each parent coach and participant. This is slightly less than the 18 contacts that were part of the original structure of the training program (weekly contacts for the first 3 months; once a month contact for months 4–9). Parent coaches reported connecting with parents via text message the most often (1536/2262; 67.9% of contacts), followed by emails (423/2262; 18.7% of contacts), phone calls (251/2262; 11.1%), in-person visits (50/2262; 2.2% of contacts), and other methods (video calls; 2/2262; 0.09% of contacts). Thirty-nine participants met in person with their parent coach at least one time (34%).

There was more frequent contact in the first 3 months as compared to months 4–9. Seventy percent of the phone calls occurred in the first 3 months (175 vs. 76). There were also more texts and emails in the first 3 months (texts: 951 vs. 586; emails: 375 vs. 48). Parent coaches reported that they referred 47.0% of participants back to the medical team in response to a question raised during a coach–participant contact, and 24.3% of participants were referred to the medical team more than once.

Coaches completed 98% of assigned monthly fidelity monitoring procedures. Of the 116 participants who were matched with a parent coach, 78% reported at least monthly contact with their parent coach across the 9-month intervention, with 87% reporting at least one contact with their parent coach through the final month of the intervention period. Parent coaches reported their most discussed topics were supporting the parent and child’s adjustment to diabetes, daily diabetes management, and discussing eating/dietary choices.

Ninety-two percent (N = 34) of coaches completed satisfaction questionnaires at the end of their involvement with this study. Coaches reported high satisfaction with their participation in the project, and 94.1% stated they would recommend the program to others (Table 2).

More than 90% reported that the training and monthly supervision calls were very or somewhat helpful. More than half (19 total, 55.8%) of the coaches noted a sizeable increase in their confidence to manage their own child’s diabetes during the project. Parent participants reported that their parent coaches were helpful (83.6%) and a good listener and supportive (89.0%, see Baudino et al., 2023 [28]).

In the interviews (N = 7), parent coaches reported on their experience of the trial, as well as ways to make improvements in future similar programs. See Table 3 for the themes and example quotes.

Interview data from parent coaches included themes about the elements of the parent coach program and the elements of the parent coach/parent participant relationship.

Parent coaches shared that the training and the supervision calls from the study staff were helpful and acceptable, with specific appreciation for the skill rehearsal as part of training. Parent coaches stated that the structure and guidelines given for the frequency of contacts, while also remaining flexible, was generally acceptable. However, they acknowledged that getting participants to engage regularly was hard at times. They discussed that getting no response from their participants was hard for them to understand. Parent coaches proposed ideas like having a standing time in their own schedule (e.g., “office hours”) or ways that the study team could prepare participants more for the intervention prior to making assignments. Parent coaches reported feeling more affectively connected to their participants when they had in-person meetings or phone calls compared to other communication methods yet appreciated the flexibility and instrumental utility of being able to have a quick text conversation about a specific issue. Coaches shared thoughts on expanding the program to adding parent coaching when children start new diabetes technology.

Parent coaches stated that establishing rapport and building a warm and trusting relationship with their participants was gratifying for them, and they speculated that this was likely the most helpful portion to participants. They emphasized that they had the lived experience which translated to them being able to share information and support more generally. They noted that they felt most helpful to parents who were matched to them based on their experiences or characteristics (e.g., father parent coach with a father parent participant) or characteristics of their children (e.g., matching on child age at diagnosis). Parent coaches stated that they wished they had access to a program like this when their own child was first diagnosed and that they participated in this parent coach program as a method of giving back. Some parent coaches noted that there were emotionally difficult moments for themselves during their coach experiences, especially when the families they were matched with experienced challenges, such as a parent coach who worked with a parent who did not realize that there was no cure for T1D. Parent coaches felt gratified by their participation in the program and reported that they established strong friendships and connections with their parent participants. Parent coaches generally felt that their participants both benefited and enjoyed the intervention. Many stated a sense of noticing their own personal growth while being a parent coach and increased confidence in their diabetes knowledge. They also reported that the coaching experience afforded them the opportunity to reflect on their own change in knowledge or attitude since their own child was diagnosed and that becoming aware of their growth led to positive feelings.

## 4. Discussion

This paper shared the activities and experiences of the parent coaches in the First STEPS study. Overall, the process of training and supporting our coaches resulted in parent coach experiences that were enjoyable, meaningful, and feasible for parents of children diagnosed with T1D. The findings reported in this paper move the field forward in understanding the experience of peer coaches in providing support for parents of newly diagnosed young children with T1D.

A core goal in creating a peer support relationship is to facilitate a trusting relationship. The exact frequency and structure of each peer mentorship program will vary depending on the specific aims and logistics of the project. Though there were slightly fewer total contacts than the guidelines instructed during training, there is support for the idea that the First STEPS program was successful in supporting the establishment of trusting relationships. Parent coaches reported regular reciprocated contact with their parent participant through the end of the 9-month study, and there was high satisfaction reported from participants and coaches. Prior research in health behavior mentoring has emphasized the importance of a good match or fit between coach and participant [29], though there is limited understanding on which characteristics matter most in forming a successful match. Future studies should explore more about the relative importance of demographic characteristics, personality characteristics, and individual mental health and family medical history in creating pairs.

Both participants and parent coaches reported high satisfaction with the intervention. Most participants reported their parent coach was helpful, and parent coaches reported that they perceived their contacts with participants as moderately helpful. In this way, participants reported a perception that parent coach contact was more helpful than the parent coaches did. One possible explanation is that parent coaches’ personality types were such that they underestimated their impact and felt there was always more that they could have done. Another explanation is that parent coaches had the benefit of years of experience in family-based management of T1D. This might have afforded the parent coaches the perspective to be selective about what portions they were sharing with their participants; however, this same awareness would not be possible for the participants. In future studies, we recommend asking parent coaches what they would think would maximize their helpfulness to understand this phenomenon further.

Parent coaches also reported that the study training and supervision were acceptable and useful to prepare for their role. They generally spent less than 1 h per week on the study over time. Though in general the interactions were minimally challenging, there were some difficult moments when parent coaches were worried about their participants or participants’ children or they re-experienced uncomfortable memories from their own family’s history. The licensed mental health professional is a critical leader of the team to provide individualized and responsive support for parent coaches in these scenarios.

Many coaches reported that their confidence to manage their own child’s diabetes increased over the project. Prior research has demonstrated the increase in self-confidence for peer coaches, noting that the mechanism is likely through coaches experiencing personal mastery and especially when they feel they have made a positive contribution [30]. Parent coaches also reported the sense of increased confidence and knowledge to manage their own child’s diabetes and awareness of their own personal growth since their child’s diagnosis of T1D. These changes might also be related to parent coaches receiving regular guidance and support from the parent coach liaisons on the research staff.

There were some limitations to this study. Many coaches had similar identities and backgrounds to one another (e.g., gender, race/ethnicity, culture, education level, marital status), thus limiting the transferability of these results to other groups. It is possible that there were features of the study protocol (e.g., time of training, data collection procedures, incentive structure, recruitment practices) that could have been biased against or less appealing to individuals from different backgrounds. We would recommend working with members of these other communities not represented in our sample to identify any potential barriers to participating as a coach. There was a limitation with how the data were collected with regards to texts/emails, where parent coaches only recorded any communication that was reciprocated or returned. The interview data suggested that parent coaches reached out in line with the guidelines from this study, yet parent participants responded at a lower rate. Parent coaches also used their own technology, time, and space to engage in contacts with participants and reported back to the study team about contacts over the past month. An alternative approach might be providing study accounts, phones, technology, or common spaces to permit for closer monitoring by the research team. This could have an additional benefit of removing any logistical barriers related to technology between coaches and participants, though none were reported in this study. Alternately, use of a specific phone application, or device, could have also interfered further with the ease of communication. In this study, the decision was made to permit parent coaches to use their own devices and accounts in order to provide a more naturalistic connection with the parents.

There may be benefits to future parent coach peer support interventions from the findings in this paper. There were some elements unmeasured in First STEPS that may be theoretically impactful in a peer-to-peer support model, including the personality types of coaches or the presence of psychiatric or neurodevelopmental diagnoses in the family system of the participant that might impact the parent–child relationship. Future studies could also explore if peer coaching can help buffer any of the known family functioning difficulties that parents of young children with T1D experience [31]. We did not measure any changes in marital satisfaction or status for participants, and this could be an area for future study. Future parent coach programs may benefit from facilitating connections between coaches and participants during hospital stays or clinic visits to try to mitigate communication barriers. The coach role in this study was as a research volunteer who received a small gift card per participant and who averaged less than 1 h/week working on the study. There could be alternative models such as hiring parent coaches as hospital employees as was described by Englander et al. (2020) in a substance use disorder program [32]. In that model, the coach is integrated into the medical team and paid for their time, which could result in increased time with each family.

## 5. Conclusions

There is evidence in support of the procedures used in this study to provide parent coaching, or peer mentorship, to parents of young children after initial T1D diagnosis. It might be that a modified schedule for parent coach contact with participants should be offered, with an every-other-week schedule in the initial period after diagnosis, instead of weekly, to better match what participants responded to. There were generally high levels of feasibility and satisfaction, both from coaches and parent participants in the trial.

## Figures and Tables

**Table 1:** Sociodemographic characteristics of parent coach participants.

Measure	M (SD) or Frequency
Age	37.9 ± 3.9
Gender, female	35 (94.6%)
Relationship to child	
Mothers	35 (94.6%)
Fathers	2 (5.4%)
Marital status, married	31 (83.8%)
Education	
12th grade	1 (2.7%)
Partial college	6 (16.2%)
2-year college	2 (5.4%)
4-year college	13 (35.1%)
Graduate/professional training	15 (40.5%)
Work schedule	
Work full-time	17 (46.0%)
Work part-time	4 (10.8%)
Do not work outside the home	11 (29.7%)
Student	1 (2.7%)
Flexible schedule	4 (10.8%)
Household income	
USD 40,000–64,999	1 (2.7%)
USD 65,000–99,999	8 (21.6%)
USD 100,000–199,999	19 (51.4%)
USD 200,000 and above	6 (16.2%)
Decline	3 (8.1%)
Race/ethnicity	
Asian American	1 (2.7%)
Black or African American	2 (5.4%)
White, non-Hispanic	30 (81.1%)
Hispanic/Latino/a/x	3 (8.1%)
Not reported	1 (2.7%)
Age of child at T1D diagnosis, years	3.6 (1.5)
Age of child with T1D when consented as a parent coach in the study	7.5 (2.1)
Child T1D duration in years	3.8 ± 2.3
Child currently using CGM	81.1%
Duration as parent coach, days	639 ± 260

Note: N = 37.

**Table 2:** Parent coaches’ satisfaction and feedback on the study (N = 34).

Item/Measure	Mean/Frequency
Summarized from monthly surveys (1, least—5, most)	
Helpfulness of contacts	3.84 ± 0.57
How much they perceived participants enjoyed contacts	3.69 ± 0.53
How challenging interactions were	1.52 ± 0.65
Overall End of Study Data	
Satisfaction with being a parent coach	
Very satisfied	30 (88.2%)
Moderately satisfied	4 (11.8%)
Usefulness of the training	
Very helpful	31 (91.2%)
Moderately helpful	3 (8.8%)
Usefulness of monthly supervision calls with study staff	
Very helpful	17 (50.0%)
Somewhat helpful	15 (44.1%)
Neutral	2 (5.9%)
Would recommend to others (Yes)	32 (94.1%)
Change in confidence to manage DM during project	
Much more confident	5 (14.7%)
Slightly more confident	14 (41.2%)
No change	14 (44.1%)
Most preferred method of communication	
Phone	8 (23.5%)
Text	20 (58.8%)
Email	3 (8.8%)
Combination	3 (8.8%)
Weekly time spent on the project	
Less than 1 h	25 (73.5%)
1–2 h	9 (26.5%)

**Table 3:** Themes from parent coach qualitative interviews (n = 7).

Theme Name	Example Quotes
Elements/structure of the program coach program	The thing that made it hard was timing. I know that it just got harder to put 30 min of sitting down. I know this sounds crazy to some people. But yeah, once [the participant] and I sort of picked like one day a week if she needed to touch base, she could reach out to me. We just kind of had a window sort of standing. I wish [hospital] did was that every time a family starts a new medical device, I wish you were connected with a family that’s on that device and that has been on that device a couple of years… it is so overwhelming learning the new technology. You learn so much through other people more so than you do in any of the trainings that you go to.
Frequency of contact	[The first participant], I would reach out a lot in the beginning and she doesn’t really need it. But it’s fine. She didn’t complain. The [second participant], even after our first three months, we were still communicating pretty regularly. But as far as the guideline, I think it works because it would sort of hit those groups. Just that I don’t know that I would want to email, or text, or call every four weeks or so [so consider reducing contacts in the later months]. I think saying, hey, I understand it’s really tough to adjust to this new normal. You have another six months in the program. Please at any time feel free to give me a call or a text, if people haven’t been responsive at all.
Support from parent coach supervisor	I definitely think the [supervision calls] checking in monthly was really good because, as the person initiating, you feel like you don’t want to go in empty-handed honestly… so you make additional effort. I relied on [supervisor’s name] especially when things were so stressful with [participant’s name]. I remember waiting to hear back from her, wondering, worrying if things were going okay. I picked up the phone and called [supervisor] and it was helpful to talk through.
Modes of communication	I mean the phone calls were great. They were very much where we connected on the phone and had a good conversation. Texting was definitely for a specific question on equipment and types of insulin and things like that where the phone communication was more of a talk about what in life is going on. I think just maybe half of the communication be either by phone call talking or by meeting in person, which is probably not going to happen a lot these days. I get that. And we live off so far away. But FaceTime, I wish I would have done that more. If I can go back and change it, that’s one thing I would definitely change. I should have met her. I think it would have been better. But there was never a good time.
Relationship building with parent/coach	I think the connections were really powerful. They’re really powerful. When you’re able to establish a relationship, it’s a powerful thing. The one thing in general that I noticed from my experience is a lot of them don’t want to know what you know. They just want to know that you understand and that you’re there for them. My first [community] support group [outside of the First STEPS program] was all about their tragedies and everything, their worst nightmares. Well, that’s really not what you want to hear. You don’t want to hear like, you know what, I heard that everything is going to be perfect. Of course that’s not the truth. But you want to hear like, yes, this will be a challenging time for you but this is what you do to make it better. This is what you do to help, you’ll get through that.
Sharing expertise/learning	[Participants] understand that you actually have been through it also. So it’s not just like ‘that sucks’, but ‘that sucks, because it sucks for me too.’ I guess just being able to share some of the things that we’ve learned through experience. Things I wish I had known but I didn’t know at the time. Things that worked for us, and also to hear what was working for other people; what other people were having trouble with. Even if I’m telling them something, I’m learning as well right along with them. Even if you’re telling them something that you already know, it just rehashes it for you.
Personal growth	I think I felt that I knew more than I realized I knew. It helped me feel more confident in my own knowledge and my own strategies for caring for my daughter, yeah, because I was able to help others. I definitely have learned that it doesn’t have to be perfect. Obviously that’s hard for me. That is hard for me. Seeing other people sort of with similar struggles, I tell them you don’t have to be perfect. So I tell them they don’t have to be perfect. You realize so yourself sometimes, it doesn’t have to be perfect for you either. We made it through. Sometimes I just felt proud of myself, proud of my husband, proud of [my daughter] most importantly.
Gratification	I loved it when people were like, wow, you made their day. Like you helped me through this. It was hard. I feel like I’m doing well now. You feel like you helped to establish that sense of like, hey, I can do this. And it was also just rather fulfilling, just to be there for somebody else going through something similar. But my experience entirely is pretty amazing. It was the opportunity to give back and to be there for someone just like someone had been there for me. So I really enjoyed it.

## Data Availability

The raw data supporting the conclusions of this article will be made available by the authors on request. Sharing the data could potentially compromise the privacy of the participants, as stipulated in the ethics approval. Requests to access the datasets may be considered on a case-by-case basis, subject to review and approval by the authors and appropriate ethics board.
